# Complications of regional and general anaesthesia in obstetric practice

**DOI:** 10.4103/0019-5049.71039

**Published:** 2010

**Authors:** Ashok Jadon

**Affiliations:** Department of Anaesthesia, Tata Motors Hospital, Jamshedpur, India

**Keywords:** Complications of anaesthesia, general anaesthesia, regional anaesthesia, obstetric anaesthesia practice

## Abstract

Any anaesthetic technique, either regional or general, has potential for complications. Moreover, it has been seen that in obstetric patients, the complications are potentiated due to pregnancy-related changes in physiology and due to various other factors. Increasing trend of caesarean section in the setting of increasing maternal age, obesity and other concomitant diseases will continue to challenge the obstetric anaesthetist in his/her task of providing safe regional and general anaesthesia. This review has highlighted the possible complications of regional and general anaesthesia encountered during the obstetric anaesthesia practice.

## INTRODUCTION

Obstetric anaesthesia is generally considered to be one of the higher-risk areas of anaesthetic practice. Changes in maternal physiology during pregnancy and the care of both mother and foetus present unique challenges to the obstetric anaesthetists. Although new systems and technologies are developing to provide consistent and safe anaesthetic care to pregnant mothers, the modern-day obstetric anaesthetist has to also grapple with issues related to changing population characteristics, including maternal obesity, advanced maternal age and an increased complexity of medical diseases (including cardiac diseases), which may affect women with a reproductive potential.

Both regional and general anaesthesia carry with them the potential for complications, some of which, although rare, may be serious, life-threatening and/or permanently disabling. Complications of regional anaesthesia and general anaesthesia that are commonly encountered during obstetric anaesthesia are discussed in this review.

## REGIONAL ANAESTHESIA IN OBSTETRICS

In Great Britain, a number of high-profile legal cases in the 1950s concerning major complications of neuraxial techniques led to its decline for more than two decades.[1] However, over the last 30 years, the use of regional anaesthesia is rapidly increasing. One study from the UK has shown that the rate of regional anaesthesia for elective caesarean section (CS) rose from 69.4% in 1992 to 94.9% in 2002, where spinal anaesthesia was used for 86.6% of the cases.[2] Various factors like improved maternal and foetal safety with regional anaesthesia[3] and confidential enquiry into maternal deaths due to general anaesthesia have been responsible for the increased use of regional anaesthesia.[4] Although serious complications are uncommon with regional anaesthesia, they must be considered and should be discussed with the patient.

## COMPLICATIONS OF REGIONAL ANAESTHESIA

### (A) Complications with central neuraxial blockades

Central blockades provide excellent labour analgesia and safe anaesthesia for CS and are associated with a low incidence of severe complications. The following complications can occur with central neuraxial blockades (CNB).

Post-dural puncture headache (PDPH): PDPH is a common complication of neuraxial blockade.[5] Parturient constitutes the highest risk category, the reported incidence in these patients varying between 0 and 30%.[6] PDPH is related to the size as well as the type of the spinal needle used, and it is progressively reduced with the use of thinner Quincke-type spinal needles.[7] Pencil point needles have a lower incidence of PDPH than cutting needle tip designs.[8] PDPH is a complication that should not be treated lightly. There is the potential for considerable morbidity due to PDPH.[9] It is reported that untreated PDPH leads to subdural haematoma[10] and even death from bilateral subdural haematomas.[11] Therefore, anaesthesiologists are advised to prevent PDPH by optimizing the controllable factors like spinal needle size as well as shape while conducting spinal anaesthesia.[12]

PDPH is usually self-limiting and spontaneous resolution may occur in few days. Therefore, the authors recommend approximately 24 h of conservative therapy. Various pharmacological (e.g., Methylxanthines, ACTH, Caffeine) and interventional measures (e.g., epidural saline/dextran) are available to treat PDPH; epidural blood patch (EBP) has a 96–98% success rate and has been recognized as the definitive treatment for PDPH.[13,14] Prophylactic EBP is also gaining acceptance.[15]

Neurological complications[16]: Serious neurological complications related to regional anaesthesia are, fortunately, very rare. The incidence of permanent or transient neurologic complications after CNB is estimated to be between 1/1,000 and 1/1,000,000. Direct trauma to the nervous tissue may occur at the level of the spinal cord, nerve root or peripheral nerve. The epidural needle or spinal needles may touch the nerve roots or may directly injure the spinal cord. Scott and others, monitored 505,000 epidural blocks in parturients, finding only 38 single-root neuropathies (0.75/10,000). Cauda equina syndrome is another annoying complication of CNB. Rigler and others, postulated that the combination of trauma, maldistribution and a relatively high dose of local anaesthetic resulted in this neurotoxic injury.[17]

Epidural abscess is a rare but dreaded complication of CNB. Epidural abscess is usually due to infection in the body seeding the epidural space. In one review, epidural anaesthesia was associated with only one in 39 epidural abscesses.[18] Neurologic deficits will progress as the spinal cord is compressed. Other symptoms include lower extremity pain, weakness, bowel and bladder dysfunction and paraplegia. Urgent surgical treatment is necessary.

Epidural haematoma: The literature has shown that epidural haematoma is another feared, but rarely seen, complication of regional anaesthesia (1/150,000–250,000) in healthy patients.[19,20] Most epidural haematomas following regional anaesthesia occurred in patients with haemostatic abnormalities, particularly those on anticoagulants. Low-molecular weight heparins have been responsible for over 35 epidural haematomas following regional anaesthesia, and should be considered a strong relative contraindication. The current evidence suggests that a platelet count of more than 80 × 109/L is adequate for the administration of neuraxial anaesthesia provided that there are no additional risk factors. A recent survey[21] confirmed that 64–78% of the units were willing to administer neuraxial anaesthesia if the platelet count was 80 × 109/L or above.

#### Cardiovascular complications

Hypotension: Hypotension following neuraxial blockade is due to sympathetic inhibition, which causes a significant decrease in the venous return due to dilatation of the resistance and capacitance vessels.[22] Hanss and others, have identified an interesting use of heart rate variability technology to potentially prevent this problem.[23] Pre-load with crystalloids[24] to prevent hypotension is controversial as it induces atrial natriuretic peptide secretion, resulting in peripheral vasodilatation and hypotension.[25] A more rational approach is coloading, i.e. giving fluid during the procedure.[24] Ephedrine has been recommended as the vasopressor of choice for the hypotensive obstetric patient.[26] However, evidence-based analysis has shown that ephedrine and α-adrenergic agonists (phenylephrine) appear to be equally efficacious.[27]

Bradycardia: Decreased pre-load after spinal anaesthesia initiates reflexes that cause severe bradycardia. Atropine is typically used as the first line of therapy and also for prophylaxis.[28]

Supine hypotensive syndrome of pregnancy: Sometimes, severe syncope may occur along with hypotension and bradycardia due to reflex cardiovascular depression. The cause was identified as compression of the inferior vena cava by the gravid uterus, reducing the venous return and right atrial pressure.[29,30]

Cardiac arrest:[31] Cardiac arrests occur significantly more often following spinal anaesthesia compared with after epidural anaesthesia. An overall incidence of seven cases of cardiac arrest for every 10,000 spinal anaesthetics versus one case for every 10,000 epidural anaesthetics has been reported. Three possible mechanisms, e.g. respiratory, cerebral and circulatory, have been speculated for cardiac arrest during neuraxial anaesthesia. Greater sedation has been observed with high spinal blocks. The possible mechanisms are the rostral spread of local anaesthetic agents or a reduction in the function of the reticular activating system caused by an interruption of the afferent inputs. There is some evidence in the early literature that cerebral hypoxia might occur during spinal anaesthesia in some patients. A circulatory etiology for cardiac arrest during spinal anaesthesia is directly or indirectly related to the blockade of sympathetic afferents and decrease of catecholamine release by the adrenal medulla.[22,23]

Extensive block:[13] This is unusual with an intentional subarachnoid block unless there has been an inappropriately high dose of local anaesthetic or previous failed attempts at epidural placement. However, it may occur with normal dosage also due to rostral spread of anaesthetic drug. Initially, it was thought that increased pressure in the epidural space can compress the subarachnoid space, thereby disseminating the local anaesthetic. Recent research has shown that it is due to a hormonal (progesterone) effect. Subdural or subarachnoid blocks can happen unintentionally during epidural placement, causing an accidentally high block.

Shivering: The incidence is 20–70% in women receiving neuraxial blockade for labour or CS. This incidence is more in spinal anaesthesia than in epidural anaesthesia.[13]

Backache: Back pain in women during pregnancy is up to 76%. Previous studies reported that epidural anaesthesia for labour and delivery was associated with long-term backache. However, randomizes controlled trials and prospective cohort studies have convincingly proved that new, long-term, post-partum back pain is not caused by intrapartum epidural analgesia.[32]

Catheter breakage: Epidural catheters may rarely break or shear. If part of a catheter is left in a patient, the patient should be informed. However, no surgery or attempts to retrieve the catheter are warranted unless there are persistent neurologic symptoms.[33]

Local anaesthetic convulsion:[34] Convulsion occurs when the critical brain tissue concentration of local anaesthetic is exceeded. Invariably, this happens with accidental intravascular injection. The previously reported incidence was 0–0.5%, whereas it is now one in 5,000–9,000. Prompt recognition and management is essential for better prognosis.

Miscellaneous:[35] The incidence of paresthesia is 8.5–42% and incidence of intravascular cannulation or blood vessel trauma is 4–12%. The incidence of inadequate analgesia in uniport catheters ranges from 31 to 32.7% and for the multiport catheters from 11 to 21.2%.

### (B) Complications with non-central blockade regional anaesthetic techniques

These techniques are to be employed when the facilities for central blockade are not available or CNB is contraindicated. Anaesthetic complications are direct injury to the mother/foetus or toxicity of the local anaesthetic agent.[36]

Complications of general anaesthesia: General anaesthesia may lead to loss of airway control, with anoxia and aspiration of gastric contents. This risk associated with obstetric general anaesthesia has led to regional techniques being used wherever possible. General anaesthesia is now used mainly for true emergency cases where there is insufficient time for a regional technique.[37] However; general anaesthesia has the advantage of rapid induction, less hypotension, cardiovascular stability and better control over airways and ventilation. The major concerns regarding the use of general anaesthesia for the obstetric population are difficulty in airway management (failed intubation) and acid aspiration. Awareness and drug toxicity are few other complications associated with general anaesthesia.

**Figure 1:**
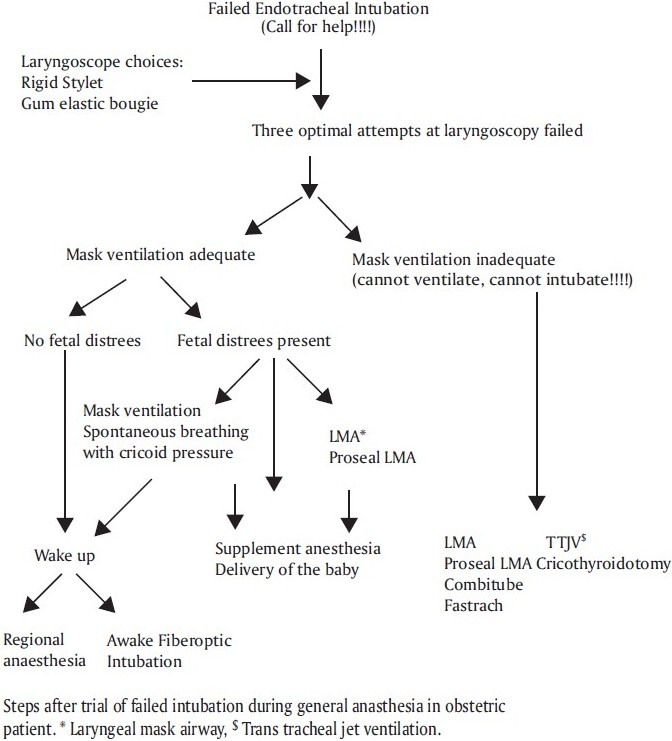
Algorithm for difficult intubation

Failed intubation:[38] The incidence of difficult obstetric intubation has been quoted 1:30. It is well known that pregnant women and women giving birth offer special anaesthetic challenges, where a four to five-times higher frequency of intubation problem and faster development of hypoxia are the main cause of mortality. However, the characteristics of pregnant women have changed, whereby average maternal age, body mass index and number of comorbidities have all risen. These factors have significantly contributed to a rising CS rate and may also affect the risk of difficult/failed intubation.[39] Circumstances like emergency CS may also lead to an inadequate airway assessment, which can contribute to the risk of difficult or failed intubation. When general anaesthesia is to be used in obstetrics, the method of airway management will depend on the urgency of the procedure and the anticipated ease or difficulty of intubation and ventilation. All equipment for routine and emergency airway management should be immediately available [Table 1]. If tracheal intubation is unsuccessful in the first attempt, steps outlined in the difficult airway algorithm should be initiated [Figure 1].

**Table 1:** Equipment for airway management in obstetrics

Routine
Laryngoscope, multiple blades (Mac 3,4, Miller 2,3)
Endotracheal tube (5.0-7.0)
Oral airways (80, 90 100 mm)
Nasal airway (7,8,9,)
Laryngeal masks (size 3 and 4)
Combitube, Stylets and bougie
Emergency
Tube exchanger
Cricothyrotomy kit
Transtracheal jet ventilation equipment
Light wand, retrograde intubation equipment
Anticipated difficult: non emergency airway
Fiberoptic laryngoscope and accessory equipment/ medication
Fixed fiberoptic blades (Bullard, Wu scope, Upsher)

Pulmonary aspiration of the gastric contents: Data from the United States reveal that aspiration of gastric contents is still a leading cause of maternal anaesthetic death, accounting for 33% of the fatality. The risk of aspiration is high in obstetric patients due to physiological changes (decreased gastric emptying, increased gastric volume, decreased pH and decreased gastroesophgeal sphincter tone), obesity and labour-induced nausea and vomiting.

Prevention of pulmonary aspiration in obstetric patients: The first step towards prevention is to recognize that every parturient is at risk of aspiration. As aspiration is commonly associated with general anaesthesia, if possible, GA has to be avoided. If GA is unavoidable, the following preventive strategies may be adopted[40]:

### Position during induction

Some authors recommend a head-up position. But, the best position is the sniffing position, and the head is supported on a small pillow.

### Prevention of muscle fasciculation

Early belief that muscle fasciculation with the use of succinylcholine causes an increase in the gastric pressure and predisposes gastric regurgitation is debatable. It is suggested that it is not a serious concern as fasciculation is weak in pregnant patient and that it also increases the tone of the lower oesophageal sphincter thus preventing passive regurgitation.

Cricoid pressure (Sellick’s maneuver): Cricoid pressure has been shown to be effective in preventing the reflux of gastric contents into the posterior pharynx even with high gastric pressure up to 50–94 cm H_2_O.

Prevention of difficult airway complications: Increasing evidence has shown that inability to control airway during emergency induction with GA is associated with aspiration. Therefore, early recognition (anticipation) of airway difficulty, proper planning and its execution is crucial to prevent aspiration.

Awake extubation: Leaving the endotracheal tube in place till the patient is fully awake and completely responsive to commands and has no sign of muscle weakness is mandatory to avoid aspiration.

Fasting order: Practices vary from institute to institute due to various scientific as well as social reasons. The consensus is to continue the practice of 6-h fasting, with the exception for patients in whom epidural is working well (such patients can be allowed to have clear fluids orally, about 100–200 ml/h).[39]

Prokinetics and anticholinergics: Dopamine antagonist (metoclopramide) 10 mg PO/IM 1 h before induces gastric emptying and, with H_2_ blockers, it increases the gastric pH above 2.5 and volume <25 ml. Anticholinergics helps in reducing the gastric acid volume and may be used as combination therapy.

Antacid prophylaxis: A dose of 30 ml of 0.3 M sodium citrate has been a standard for three decades. Sodium bicarbonate 8.4% (20 ml) has also been suggested. However, this is associated with excess gas production. Most commonly used antacids are H_2_ blockers and proton pump inhibitors. Cimitidine 300 mg and Ranitidine 150 mg when given orally the night before surgery and 1–2 h before anaesthesia are effective in reducing the volume and increasing the pH. Famotidine 20 mg (PO 2 h before or IM 1 h before) or Nizatidine 150–300 mg PO will raise the pH >5.7 and decrease the volume up to 10 ml. Omeprazole 20–40 mg PO and Lansoprazole 15–30 mg PO are also effective.

Awareness: A high incidence of awareness has been reported after GA in the obstetric population. It is necessary to avoid awareness during anaesthesia as it may lead to long-lasting psychological symptoms. Use of inhalational anaesthetic agents (halothane 0.5%, isoflurane 0.6% and sevoflurane 1%) with 50% nitrous oxide virtually prevents the awareness.[41]

Way forward: Preparing a woman for emergency CS is a multidisciplinary task involving multiple steps and processes, including the timely assembly of staff. Good communication among obstetricians, midwives and anaesthetists and early involvement of senior staff for high-risk cases will surely decrease the complications. The ASA Task Force on Obstetric Anaesthesia has also given guidelines to ensure a standard care for obstetric patients and to avoid ambiguity in management plans for better outcome.[42]

## References

[1] Cope RW (1995). The Wooley and Roe Case. Anaesthesia.

[2] Jenkins G, Khan M (2003). Anesthesia for Caesarean section: a survey in a UK region from 1992 to 2002. Anaesthesia.

[3] Yantis S, Hirsch N, Smith G (2004). Anesthesia and Intensive Care.

[4] Cooper G, McClure J (2005). An extract from Why Mothers Die -, the Confidential Enquiries into Maternal Deaths in the United Kingdom. Br J Anaesth.

[5] Choi PT, Galinski SE, Takeuchi L, Lucas S, Tamayo C, Jadad AR (2003). PDPH is a common complication of neuraxial blockade in parturients: a meta-analysis of obstetrical studies. Can J Anaesth.

[6] Spencer HC (1998). Postdural puncture headache: what matters in technique?. Reg Anesth Pain Med.

[7] Halpern S, Preston R (1994). Post dural puncture headache and spinal needle design. Anesthesiology.

[8] McConachie I, McGeachie J, Thomas EJH, Peter JC (1995). Regional anaesthetic techniques. Wylie and Churchill-Davidson’s A Practice of Anesthesia.

[9] Eerola M, Kaukinen L, Kaukinen S (1981). Fatal brain lesion following spinal anaesthesia. Report of a case. Acta Anaesthesiol Scand.

[10] Zeidon A, Farhat O, Maaliki H, Baraka A (2006). Does PDPH left untreated lead to subdural haematoma? Case report and review of the literature. Int J Obs Anesth.

[11] Grieff J, Cousins MJ, Nimmo WS, Row Botham DJ, Smith G (1994). Sub-arachnoid and extradural anaesthesia. Anaesthesia.

[12] Gunadyn B, Karaca G (2006). Prevention strategy for Postdural puncture headache. Acta Anaesthesiol Bel.

[13] Bromage PR, Hughes SC, Levinson G, Rosen MA (2002). Neurologic Complications of Regional Anaesthesia for Obstetrics. Shnider and Levinson’s Anesthesia for Obstetrics.

[14] Gielen M (1989). Postdural puncture headache. A review. Reg Anesth.

[15] Cheek TG, Banner R, Sauter J, Gutsche BB (1988). Prophylactic extradural blood patch is effective. Br J Anaesth.

[16] Zakowski MI, Norris M (1999). Postoperative complications associated with regional anesthesia in the parturient. Obstetric Anesthesia.

[17] Rigler ML, Drasner K, Krejcie TC, Yelich SJ, Scholnick FT, DeFontes J (1991). Cauda equina syndrome after continuous spinal anesthesia. Anesth Analg.

[18] Baker AS (1975). Spinal epidural abscess. N Engl J Med.

[19] Horlocker TT (1996). Regional anesthesia and analgesia in the patient receiving thromboprophylaxis. Reg Anesth.

[20] Douglas MJ, Halpern SH, Douglas MJ (2005). The use of neuraxial anaesthesia in parturients with thrombocytopenia: what is adequate platelet count?. In Evidence based obstetric anesthesia.

[21] Wee L, Sinha P, Lewis M (2002). Central nerve block and coagulation: a survey of obstetric anaesthetists. Int J Obs Anesth.

[22] Baron J, Decaux-Jacolot A (1986). Influence of venous return on baroreflex control of heart rate during lumber spinal and epidural anesthesia in humans. Anesthesiology.

[23] Hanss R, Bein B, Francksen H, Scherkl W, Bauer M, Doerges V (2006). Heart rate variability-guided prophylactic treatment of severe hypotension after subarachnoid block for elective cesarean delivery. Anesthesiology.

[24] Dyer RA, Farina Z, Joubert IA, Du Toit P, Meyer M, Torr G (2004). Crystalloid preload versus rapid crystalloid administration after induction of spinal anaesthesia (Co-load) for elective Caesarean section. Anaesth Intensive Care.

[25] Pouta AM, Karinen J, Vuolteenaho OJ, Laatikainen TJ (1996). Effect of intravenous fluid preload on vasoactive peptide secretion during Caesarean section under spinal anesthesia. Anaesthesia.

[26] Burns SM, Cowan CM, Wilkes RG (2001). Prevention and management of hypotension during spinal anaesthesia for elective caesarean section: a survey of practice. Anaesthesia.

[27] Halpern SH, Chochinov M, Halpern SH, Douglas MJ (2005). The use of vasopressors for the prevention and treatment of hypotension secondary to regional anesthesia for cesarean section. In Evidence based obstetric anesthesia.

[28] Caplan RA, Ward RJ, Posner K, Cheney FW (1998). Unexpected cardiac arrest during spinal anesthesia: a closed claims analysis of predisposing factors. Anesthesiology.

[29] Lees MM, Scott DB, Kerr MG, Taylor SH (1967). The circulatory effects of recumbent postural change in late pregnancy. Clin Sci.

[30] Watkins EJ, Dresner M, Calow CE (2000). Severe vasovagal attack during regional anaesthesia for Caesarean section. Br J Anaesth.

[31] Moemen E Dynamics of Neuraxial Blocks in Obstetrics and Gynaecology. //sites.google.com/site/profezzatmoemen/dynamics-of-neuraxial-blocks.

[32] Breen TW, Halpern SH, Douglas MJ (2005). Epidural analgesia and back pain. In Evidence-based obstetric anesthesia.

[33] Mitra R, Fleischmann K (2007). Management of the sheared epidural catheter: is surgical extraction really necessary?. J Clinical Anesthesia.

[34] Rosen MA, Hughes SC, Levinson G, Hughes SC, Levinson C, Rosen MA (2002). Regional Anesthesia for labor and Delivery. Shnider and Levinson’s Anesthesia for Obstetrics.

[35] Srebrnjak M, Halpern SH, Halpern SH, Douglas MJ (2005). Epidural catheter design and the incidence of complications. Evidence-based obstetric anesthesia.

[36] Collis R, Plaat F, Urquhart J (2002). Textbook of Obstetric Anesthesia.

[37] Thomas J, Paranjothy S (2001). The National Sentinel Caesarean Section Audit Report. Royal College of Obstetricians and Gynaecologists Clinical Effectiveness Support Unit.

[38] Djabatey EA, Barclay PM (2009). Difficult and failed intubation in 3430 obstetric general Anaesthetics. Anaesthesia.

[39] Farcon EL, Kim MH, Marx GF (1994). Changing mallampati score during labour. Can J Anaesth.

[40] Cheek TG, Gutsche BR, Hughes SC, Levinson G, Rosen MA (2002). Pulmonary aspiration of gastric contents. Shnider and Levinson’s Anesthesia for Obstetrics.

[41] Wilson J (1969). Turner DJ. Awareness during caesarian section under general anaesthesia. Br J Anaesth.

[42] American Society of Anesthesiologists Task Force on Obstetric Anesthesia (2007). Practice guidelines for obstetric anesthesia: an updated report by the American Society of Anesthesiologists Task Force on Obstetric Anesthesia. Anesthesiology.

